# A Deep-Learning-Based Real-Time Microearthquake Monitoring System (RT-MEMS) for Taiwan

**DOI:** 10.3390/s25113353

**Published:** 2025-05-26

**Authors:** Wei-Fang Sun, Sheng-Yan Pan, Yao-Hung Liu, Hao Kuo-Chen, Chin-Shang Ku, Che-Min Lin, Ching-Chou Fu

**Affiliations:** 1 Department of Geosciences, National Taiwan University, Taipei City 10617, Taiwan; ttsun.sun@gmail.com (W.-F.S.); johnson606100@gmail.com (S.-Y.P.); gaassmail@gmail.com (Y.-H.L.); 2 Science and Technology Research Institute for DE-Carbonization, National Taiwan University, Taipei City 10617, Taiwan; 3 Institute of Earth Sciences, Academia Sinica, Taipei City 115201, Taiwan; backnew@earth.sinica.edu.tw (C.-S.K.); ccfu@earth.sinica.edu.tw (C.-C.F.); 4 National Center for Research on Earthquake Engineering, National Institutes of Applied Research, Taipei City 106219, Taiwan; cmlin@niar.org.tw

**Keywords:** real-time microearthquake monitoring system, deep learning, SeedLink, automated workflow, earthquake catalog

## Abstract

A timely, high-resolution earthquake catalog is crucial for estimating seismic evolution and assessing hazards. This study aims to introduce a deep-learning-based real-time microearthquake monitoring system (RT-MEMS) for Taiwan, designed to provide rapid and reliable earthquake catalogs. The system integrates continuous data from high-quality seismic networks via SeedLink with deep learning models and automated processing workflows. This approach enables the generation of an earthquake catalog with higher resolution and efficiency than the standard catalog announced by the Central Weather Administration, Taiwan. The RT-MEMS is designed to capture both background seismicity and earthquake sequences. The system employs the SeisBlue deep learning model, trained with a local dataset, to process continuous waveform data and pick P- and S-wave arrivals. Earthquake events are then associated and located using a modified version of PhasePAPY. Three stable RT-MEMS have been established in Taiwan: one for monitoring background seismicity along a creeping fault segment and two for monitoring mainshock–aftershock sequences. The system can provide timely information on changes in seismic activity following major earthquakes and generate long-term catalogs. The refined catalogs from RT-MEMS contribute to a more detailed understanding of seismotectonic structures and serve as valuable datasets for subsequent research.

## 1. Introduction

Real-time earthquake monitoring systems are essential for seismic hazard assessment, prevention, and reduction. Earthquake early warning systems, which detect moderate-to-large earthquakes (defined here as magnitude 6 and greater, *M*6+) rapidly and alert affected areas seconds before strong shaking occurs, represent a critical and practical discipline within real-time seismology [1]. Consequently, earthquake early warning systems are among the earliest developed and most widely utilized real-time earthquake monitoring systems globally [2,3], serving to mitigate hazards induced by major earthquakes, a significant concern for both society and academia. In the past decade, advancements in seismic network coverage and the application of machine learning (ML) in earthquake seismology [4,5] have enabled development of real-time microseismicity (magnitudes ≤ 5.0) monitoring systems [6]. Recently, an increasing number of ML models implemented in real-time microearthquake monitoring systems (RT-MEMS) have been developed at both global (e.g., [7]) and local scales (e.g., [8,9,10,11]).

High-resolution microearthquake catalogs furnish fundamental data essential for characterizing earthquake properties [6,12], encompassing aspects such as source mechanism, clustering patterns, nucleation processes [13,14], triggering phenomena, seismic velocity inversion, and earthquake forecasting [15,16,17]. Despite the exponential growth in seismic network data volume, a primary challenge in RT-MEMS lies in the ongoing development of novel techniques and algorithms [6]. A core objective of RT-MEMS is the implementation of robust seismic signal detection methodologies, incorporating both conventional and machine learning approaches. Among traditional algorithms, the waveform amplitude ratio of short-term average to long-term average (STA/LTA [18]) remains a prevalent technique in various semi-automated and automated earthquake monitoring systems (e.g., SeisComP3 and Earthworm [19]). Other algorithms employed in RT-MEMS include template matching for waveform similarity (e.g., [20,21]), array-based beamforming (e.g., [22]), and double-difference relative relocation (e.g., hypoDD [23]), among others. However, the increasing scale of seismic network datasets can lead to limitations in the completeness of earthquake catalogs when relying solely on traditional algorithms and their associated computational efficiency [24], potentially prioritizing larger magnitude events and their aftershock sequences [12]. Consequently, RT-MEMS utilizing machine learning models, which often detect several times more earthquakes compared to traditional methods, can generate rapid and high-resolution catalogs [6,7,8,9,10,11].

In Taiwan, the Central Weather Administration (CWA) holds the responsibility for the generation and announcement of earthquake catalogs. These catalogs are accessible via the GDMS online system (Taiwan Seismological and Geophysical Data Management System [25]) and are subject to periodic updates. A significant enhancement of the CWA earthquake catalog was implemented in 2012, involving an expansion of the seismic monitoring network and an improvement in digital resolution from 12 to 24 bits [26]. As a consequence, the annual average of recorded seismic events by the CWA increased from approximately 20,000 during the period of 1994–2011 to 40,000 during 2012–2019, with an estimated magnitude of completeness (*M*_C_) of approximately 1.0. However, due to the elevated seismicity with dozens of earthquakes exceeding magnitude 6.0 in Taiwan since 2018, the earthquake catalog after 2019 can only be complete up to a magnitude of 2 (*M*_C_ ~ 2.0). For example, considering the *M*_L_ 6.9 Chihshang, eastern Taiwan, earthquake on 18 September 2022, Sun et al. [27] generated a deep learning-based earthquake catalog, containing 14,276 events, for the two months preceding and following the mainshock. Furthermore, they selected 5691 earthquakes by 1D-hypoDD [23], manually reviewed for deep learning arrival time records, and found the *M*_C_ of this catalog reached 0.8 [27]. In contrast, the CWA catalog for the same region currently reports only 1247 earthquakes (Figure 6a in Sun et al., [27]; accessed 22 March 2023 and 24 March 2025). Even two and a half years post-event, the number of earthquakes in the CWA catalog remains significantly lower than that of the deep learning earthquake catalog, highlighting the necessity of real-time microearthquake monitoring systems (RT-MEMS) in Taiwan.

**Figure 1:**
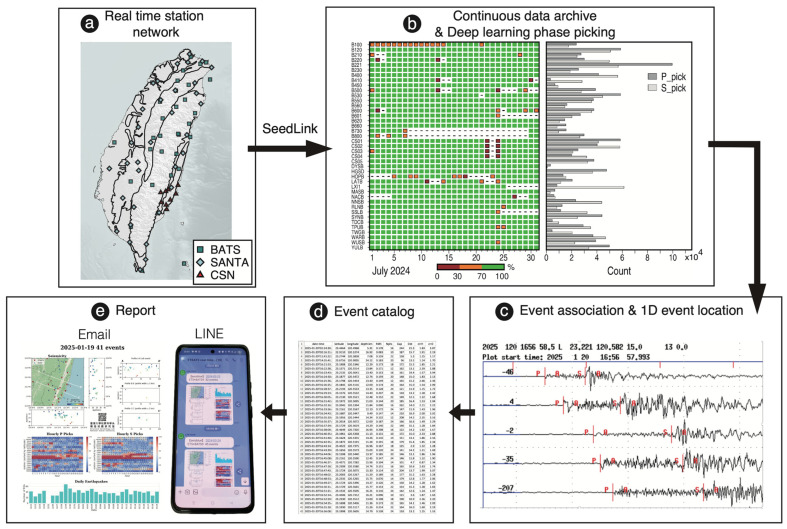
Framework of real-time microearthquake monitoring system (RT-MEMS). (**a**) Three real-time seismic networks used for RT-MEMS: BATS, SANTA, and CSN. (**b**) Contents of real-time transmitted continuous waveform data via SeedLink (**left panel**, daily completeness of each seismic station) and P- and S-wave picks (**right panel**, monthly counts) of July 2024. (**c**) Associated P- and S-wave picks of a detected and located earthquake. (**d**) Earthquake cataloging list from association step. (**e**) Earthquake reports launched via email and LINE.

We have developed a well-tested near RT-MEMS (Figure 1) for producing rapid and high-resolution earthquake catalogs in Taiwan. This RT-MEMS, implemented a modified deep learning picking model and trained by a local dataset of Taiwan, can provide a rapid and more complete earthquake catalog than the standard catalog updated by CWA. In this paper we introduce the automatic earthquake catalog producing procedure that obtains continuous database construction after receiving real-time waveform data from the permanent seismic networks, uses deep learning models to build a database of body wave arrival times, associates and locates seismic events, establishes earthquake catalogs, and generates and publishes earthquake reports. Through the three RT-MEMS established in Taiwan, we explain how to test and establish RT-MEMS and their long-term observation results for different seismic activity areas.

## 2. Data and Methods

### 2.1. Seismic Networks

**Figure 2:**
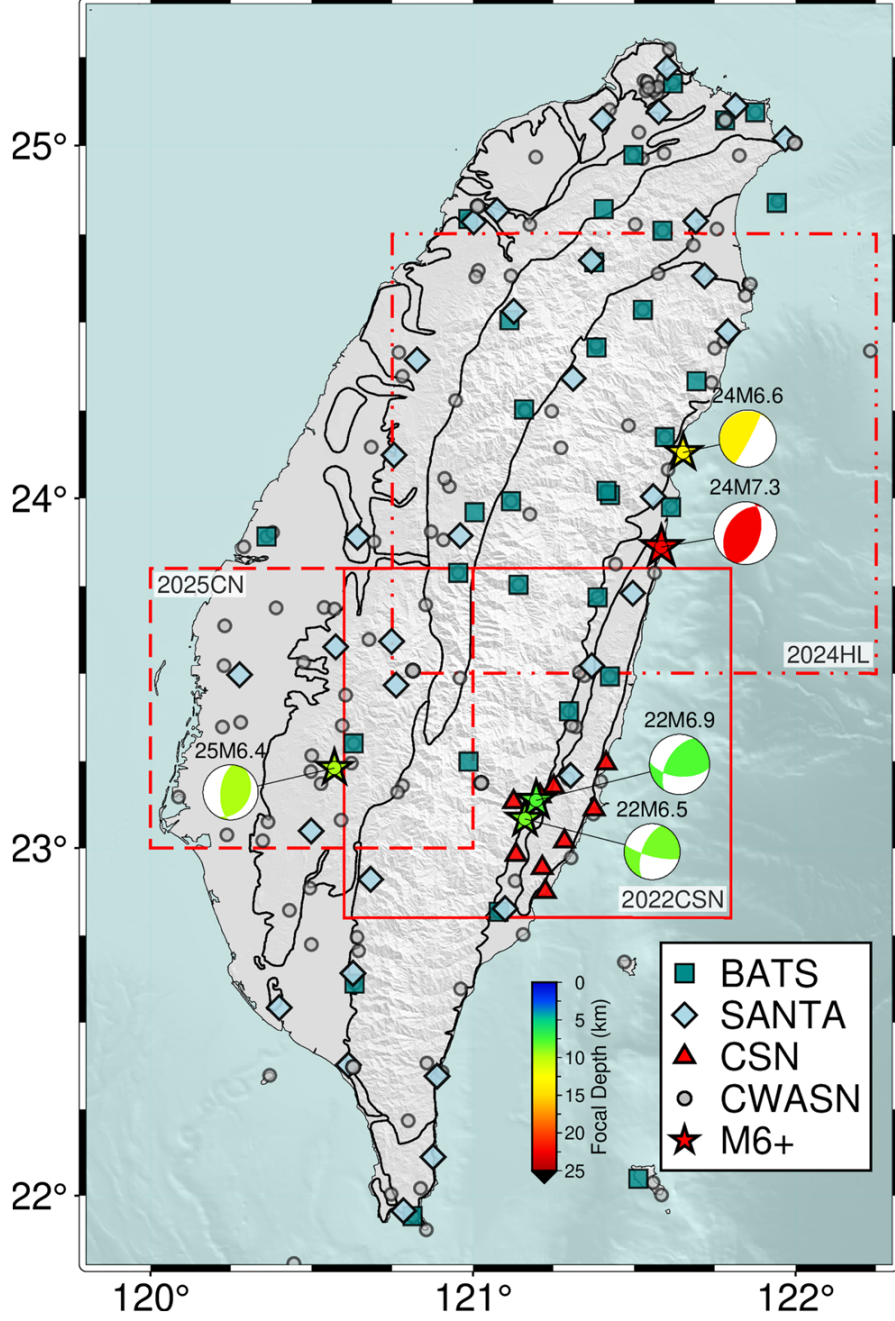
Station distribution of four permanent seismic networks, BATS, SANTA, CSN, and CWASN, in Taiwan, and locations of the three RT-MEMS. Red solid line, double-dotted-dashed line, and dashed line boxes are study areas for the 2022 Chihshang Seismic Network (2022CSN), the 2024 *M*_L_ 7.2 Hualien aftershock sequence (2024HL), and the Chia-Nan and the 2025 *M*_L_ 6.4 Dapu earthquake sequence (2025CN), respectively. The *M*6+ earthquakes (stars) locations and their focal mechanisms (beachballs) are provided by CWA and BATS, respectively, and color-coded by focal depths.

Taiwan has three island-wide permanent broadband seismic networks, the Broadband Array in Taiwan for Seismology (BATS, [28]), the Central Weather Administration Seismic Network (CWASN, [25]), and the Seismic Array of NCREE in Taiwan (SANTA, [29]), which are operated by the Institute of Earth Sciences (IES), Academia Sinica, the Central Weather Administration (CWA), and the National Center for Research on Earthquake Engineering (NCREE), respectively (Figure 2). BATS and SANTA contain 40 and 37 seismic stations, respectively, which is equivalent to CWASN’s nearly 100 broadband stations.

For monitoring microseismicity at the creeping segment of the Chihshang Fault (CSF) [30,31,32], the Department of Geoscience of National Taiwan University (NTU) and IES established the Chihshang Seismic Network (CSN), which contains five broadband seismic station in late November 2021 [27] and added four more new ones in late December 2024 (Figure 2). The real-time waveform files of CSN are transmitted to IES via 4G since June 2022. In this study, we integrate three broadband seismic networks for the RT-MEMS, including BATS, SANTA, and CSN. The continuous waveform data are transmitted from IES through the Taiwan Academic Network (TANet) to NTU.

### 2.2. Workframe of the RT-MEMS

This section describes the connection, construction, error reporting, and repair of the entire RT-MEMS, as well as the production and delivery of earthquake reports. The procedure achieved in this study for retrieving near real-time or off-line earthquake catalog includes receiving real-time waveform data from IES via SeedLink or archive, preprocessing data, detecting phase arrivals, associating and locating events, listing events into catalogs, and visualizing and publishing earthquake reports (Figure 1).

#### 2.2.1. Continuous Waveform Data

High quality continuous waveform data are the foundation of earthquake catalogs. For continuous waveform data acquisition, we use a ObsPy (Version 1.4.1) [33,34] module, SeedLink (Version 3.1) [35], to connect to the SeedLink server at IES, and request real-time continuous data stream and write received packets into directory/file structures, the SeisComP Data Structure (SDS) archive [36]. SeedLink uses the TCP/IP communication protocol to select the waveform to be obtained and specify the time period using information indicating station, channel, network, and location. Each transmission consists of an 8-byte SeedLink header and a 512-byte waveform record in miniSEED format [37,38]. One of the advantages of SeedLink is the waveform data can be retrieved even though the network has been shut down once the files were saved in the SeedLink temporal saving space. We use a ObsPy script, obspy-scan, to calculate the completeness of the continuous data, the percentage of saved waveform data points to the expected total number of data points, among seismic stations saved in the SDS archive (left panel of Figure 1b).

#### 2.2.2. P- and S-Wave Picking Database

The fundamental principle of earthquake location relies on the arrival times (seismic phase picks) of compressional (P) and shear (S) waves recorded at seismic stations after seismic wave propagation through the Earth’s subsurface. The arrival times of seismic phases observed from varying azimuths and epicentral distances provide crucial constraints for inferring the earthquake’s hypocentral coordinates. Consequently, the accurate identification and picking of P- and S-wave arrival times from continuous seismic waveform data constitute the initial and essential step in the compilation of an earthquake catalog. We implement the deep learning picking model of SeisBlue [39] into this workflow, which is modified from the model architecture of EQ-Transformer [40], the concept of noise label from PhaseNet [41], and the training framework from Generative Adversarial Network (GAN [42]), and then trained and tested on a local dataset of Taiwan. Before detecting and picking the P- and S-wave phases, we use Obspy modules to resample (to 100 Hz), detrend, demean, bandpass, and normalize the continuous waveform data. Seismic phase picking uses a 30.08-s time window, which overlap 15.04 s to the next time window, to target local to regional earthquake whose time differences between P- and S-wave arrivals on the same stations are usually between a few seconds and tens of seconds. The model determines the probability of the waveform having a P- or S-wave at each time point. When the probability predicted by the model exceeds the specified threshold (0.1–0.2), the P- and S-wave arrival times (right panel of Figure 1b) are considered to be selected and stored with related information, such as station name, phase type, and signal-to-noise ratio to MySQL (Version 9.2.0) database [43] and performed by SQLAlchemy (Version 1.4.52) [44], a database toolkit of Python (Version 3.8.10) for efficient and high-performing database access [39].

#### 2.2.3. Association

The operational concept of earthquake association involves combining all P- and S-wave arrival times within a specified time window and a defined time difference, assuming that the P- and S-waves originate from the same seismic event. The earthquake associator is adapted from PyAPA [45], whose concept was derived from PhasePAPY [46] focusing on those with similar origin times based on the S-P time estimation theory [47] (Figure 1c). We modify the processing order of candidate events, locating candidate events with more arrival times and performing parallel operations, which can be 20 to 30 times faster than PyAPA [39]. The location procedure operates in two steps: (1) candidate events with a higher number of arrival times will be located by the HYPOCENTER program [48] integrated in SEISAN (Version 13.0) [49]; (2) after removing the outliers of arrival times (stations with travel-time residuals exceeding 3 s), the candidate events that meet the location criteria (a root-mean-square (RMS) of travel-time residuals ≤ 0.5 s for each station, and location errors in depth, longitude, and latitude all ≤ 50 km) are listed to the earthquake catalog (Figure 1d). In this step, the candidate events and the location information are stored in MySQL [43] and performed by SQLAlchemy [44].

#### 2.2.4. Earthquake Report

The earthquake reports can be designed based on earthquake event conditions. For long-term seismicity observation, the reports for the 2022 Chihshang Seismic Network (CSN) contain not only daily seismicity distribution and earthquake statistics but also the hourly P- and S-wave picks, which is an indicator of whether waveform data are transmitted continuously and the background noise status of each seismic station. For aftershock observation, the reports of the 2024 *M*_L_ 7.2 Hualien and 2025 *M*_L_ 6.4 Dapu earthquake sequences focus on the short-term evolution of the seismicity in time and space. After the 2024 *M*_L_ 7.2 Hualien mainshock occurred, we increased the frequency of earthquake reports, not only daily earthquake reports, but also hourly reports during the first month, gradually changed to every four hours, then every six hours as the number of aftershocks decreased. To date, it has been changed to daily reports, and it is expected to become a background earthquake monitoring nature and conduct a long-term daily reporting mode.

The completed earthquake report is sent to the users via email and LINE [50], the most commonly used instant messaging software in Taiwan (Figure 1e). Specify the Simple Mail Transfer Protocol (SMTP) server URL and port number through smtplib, a Python suite [51], log in to the SMTP server with the email account and password, and send an email to the specified mailbox. Connect with the Line Application Programming Interface (API) through the kit requests (Version 2.31.0) [52], check the token, and send the report to the Line group as LINE Notify.

#### 2.2.5. Remediation Strategy for Automated System Failures

Power outages, network disconnections, and equipment problems may cause waveform access failures and even shut down the RT-MEMS. To date, the waveform data that SeedLink can obtain from IES is approximately up to the preceding 10 h. For automatically checking the integrity of the waveform data, we designed a program that if the real-time waveform data of more than two-thirds seismic stations in any network have not been saved later than five minutes, an email will be sent to the administrators to confirm the problem. If the problem can be resolved within 10 h, SeedLink can still automatically back up the waveform data and keep the RT-MEMS working. On the other hand, if the waveform data cannot be obtained, it will be retrieved from IES’s archive, and waveform selection and correlation will be performed manually to complete the earthquake catalog.

## 3. Applications

We have established three RT-MEMS in Taiwan for background seismicity and aftershock sequences of moderate-to-large mainshocks. The 2022CSN-RT-MEMS was built for monitoring background seismicity along a creeping fault segment in Chihshang, and the 2024HL-RT-MEMS and 2025CN-RT-MEMS for monitoring mainshock–aftershock sequences following the 2024 *M*_L_ 7.2 Hualien and 2025 *M*_L_ 6.4 Dapu earthquakes, respectively.

### 3.1. The 2022 Chihshang Seismic Network (2022CSN)

The Chihshang Seismic Network (CSN) is a part of the MAGIC-Chihshang (Multidimensional Active Fault Geo-Inclusive Observatory Center—Chihshang) project. This project aims to monitor microseismicity at the creeping segment of the Chihshang Fault (CSF) [30,31,32] and integrate measurements of radon, CO_2_, fluid chemistry, and advanced optical fiber technologies [53] to investigate fault activity and fluid movement. The overarching goal of MAGIC-Chihshang is to enhance understanding of fault behavior, crustal deformation, and fluid migration processes associated with earthquakes [54].

#### 3.1.1. Station Selection for 2022CSN-RT-MEMS

**Figure 3:**
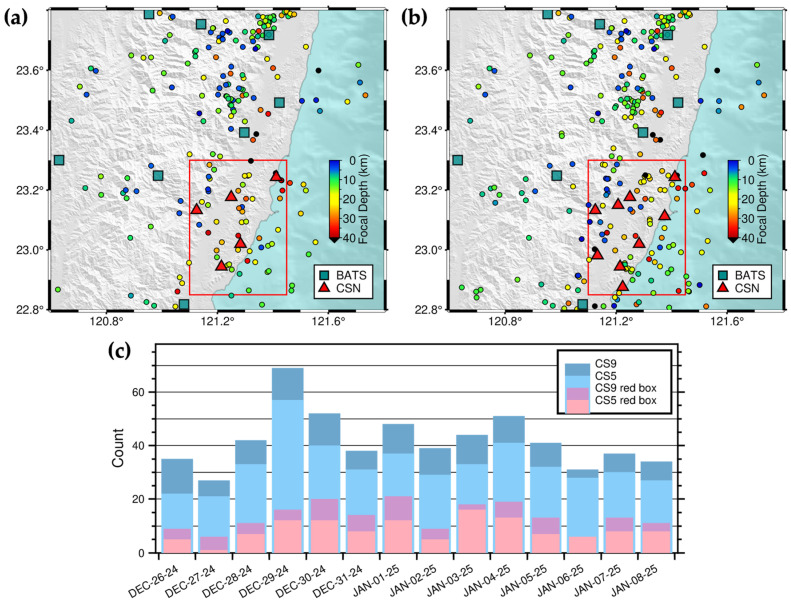
Station selection test of the 2022CSN-RT-MEMS. Two-week seismicity in Chihshang and its adjacent areas in southeastern Taiwan with (**a**) five (CS5) and (**b**) nine (CS9) CSN stations between 26 December 2024 and 8 January 2025. The red rectangles represent the Chihshang area, circles earthquakes color-coded by focal depths, and blue-green squares and red triangles seismic stations of BATS and CSN, respectively. (**c**) Daily earthquake number detected by the 2022CSN-RT-MEMS of different station combinations. The date format is month-day-year, for example, DEC-26-24 represents 26 December 2024.

Seismic station coverage is a critical factor influencing earthquake detection capability (e.g., [10]). Following the occurrence of the *M*_L_ 6.5 Guanshan and *M*_L_ 6.9 Chihshang earthquakes in eastern Taiwan on 17–18 September 2022, we established the 2022CSN-RT-MEMS. The initial five-station CSN (Figure 3a) enhanced our ability to detect earthquakes in the Chihshang area (Figure 5 in Sun et al. [27]). To further expand the monitoring area, we integrated the CSN with nine BATS stations within an 85 km radius of the center of the Chihshang area and established the 2022CSN-RT-MEMS in late March 2023.

In late December 2024, we added four new stations to the CSN (Figure 3b). A comparison of two-week earthquake catalogs (from 26 December 2024 to 8 January 2025) derived from continuous waveform data using different station configurations—15 stations (10 BATS and 5 CSN) and 19 stations (10 BATS and 9 CSN)—revealed 461 and 588 seismic events, respectively (Figure 3a,b). The addition of the four new CSN stations improved earthquake detection capability within the Chihshang area (red boxes in Figure 3a,b) and its adjacent regions (Figure 3c).

#### 3.1.2. Earthquake Report of the 2022CSN-RT-MEMS

**Figure 4:**
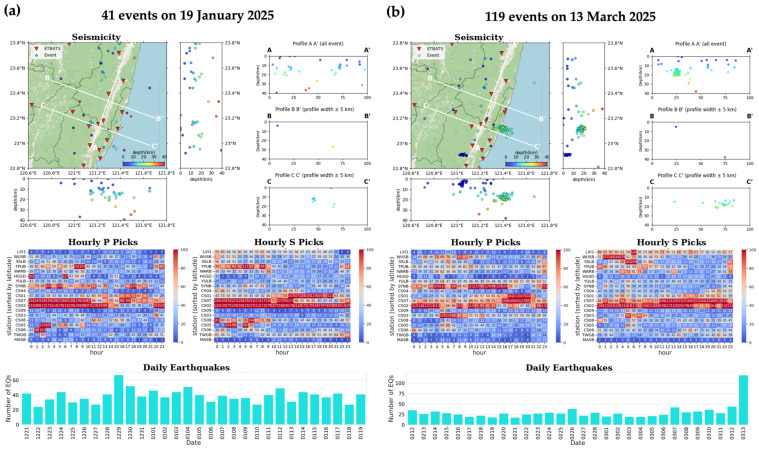
Daily earthquake reports of the 2022CSN-RT-MEMS for Chihshang and its surrounding areas in southeastern Taiwan, including seismicity in map-view and profiles, hourly number of P- and S-wave picks, and daily earthquake number for the preceding 30 days. (**a**) Daily earthquake report for background seismicity on 19 January 2025. (**b**) Daily earthquake report recording a *M*_L_ 5.7 earthquake (13:09:37 local time, UTC+08:00) and its aftershocks on 13 March 2025. In the Seismicity subplots, the red inverted triangles represent seismic stations and circles earthquakes color-coded by focal depths. The date format in the Daily Earthquakes subplots is month-day, for example, 0212 represents 12 February.

The 2022CSN-RT-MEMS generates daily reports for the Chihshang area in southeastern Taiwan. These reports include a map view of seismicity, seismicity profiles, the hourly number of P- and S-wave picks, and the daily earthquake count for the preceding 30 days. The daily report of the 2022CSN-RT-MEMS on 19 January 2025 (Figure 4a) illustrates the background seismicity of the Chihshang area (profile C-C’), and the daily report of the 2022CSN-RT-MEMS on 13 March 2025 (Figure 4b), shows aftershocks induced by an *M*_L_ 5.7 earthquake that occurred on 13 March 2025 (local time 13:09:37, UTC+08:00) at a depth of 13 km in Chengkung, about 10 km from Chihshang to the east on the coastline.

#### 3.1.3. Long-Term Seismicity of the 2022CSN-RT-MEMS

**Figure 5:**
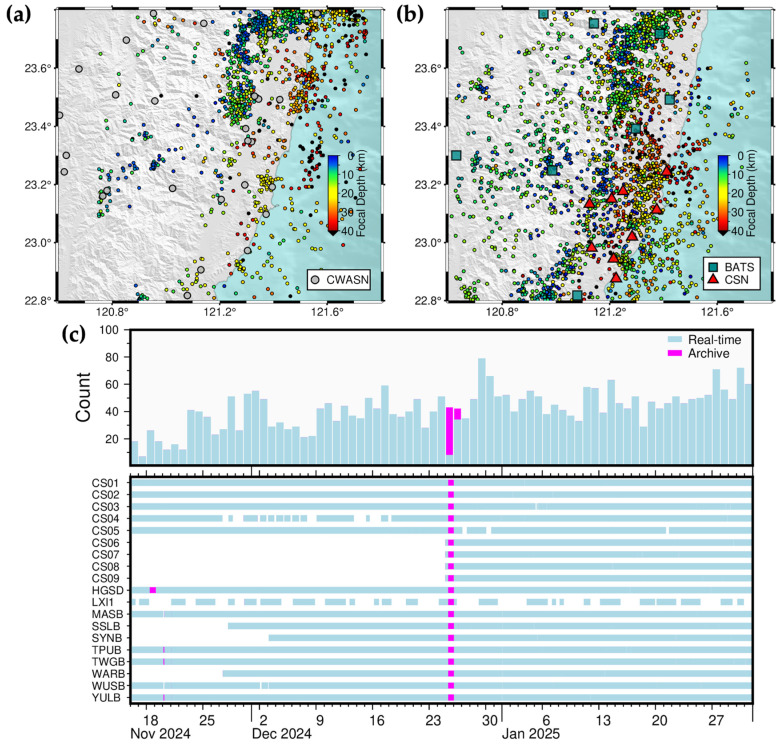
Long-term, two-and-half-month, seismicity in Chihshang area between 16 November 2024 and 31 January 2025. (**a**) 1614 earthquakes listed in the CWA catalog, downloaded on 24 March 2025; (**b**) 2722 earthquakes listed in the 2022CSN-RT-MEMS catalog, issued on 1 February 2025. The gray circles, blue-green squares, and red triangles represent seismic stations of CWASN, BATS, and CSN, respectively, and circles earthquakes color-coded by focal depths. (**c**) daily earthquake number (**upper panel**) and completeness of saved continuous waveform data (**lower panel**).

From 16 December 2024 to 31 January 2025, the 2022CSN-RT-MEMS detected and located a total of 2722 earthquakes in Chihshang and its surrounding areas (Figure 5b), whereas CWA reported 1614 earthquakes (retrieved on 10 March 2025) (Figure 5a). Because CSN contains much denser station distribution than that of CWASN, the 2022CSN-RT-MEMS does improve the ability of detecting background seismicity in the southern portion of the monitoring area (Figure 5b). During this period, a network disruption at NTU on 25–26 December 2024, interrupted real-time data transmission. Following the completion of necessary repairs, data transmission was restored, and waveform data were retrieved from IES’s data archive to ensure dataset continuity and completeness of the earthquake catalog (Figure 5c).

### 3.2. The 2024 M_L_ 7.2 Hualien Aftershock Sequence (2024HL-RT-MEMS)

The *M*_L_ 7.2 Hualien earthquake struck northeastern Taiwan on 3 April 2024, at 07:58:09 local time (UTC+08:00), causing significant damage, including 18 fatalities and 1155 injuries. Building upon experience from the 2022CNS-RT-MEMS, we established an RT-MEMS for the 2024 *M*_L_ 7.2 Hualien earthquake (2024HL-RT-MEMS) within one day, with daily reports initiated the following day. Given that the 2024 Hualien earthquake was the most significant seismic event in Taiwan since the 1999 *M*_L_ 7.3 Chi-Chi earthquake, it generated numerous felt earthquakes extending 20 km to the north. To facilitate more detailed monitoring of the evolving aftershock sequence, the reporting frequency of the 2024HL-RT-MEMS was increased to hourly and four-hourly intervals. Notably, on 22–23 April 2024, approximately 20 days post-mainshock, a sequence of three *M*6+ and 34 *M*5+ earthquakes occurred in Shoufeng, located 10 km south of the mainshock epicenter. In April 2024, CWA reported one *M*7+, seven *M*6+, and 90 *M*5+ earthquakes in the northern Hualien.

#### 3.2.1. Station Selection and Test of the 2024HL-RT-MEMS

**Figure 6:**
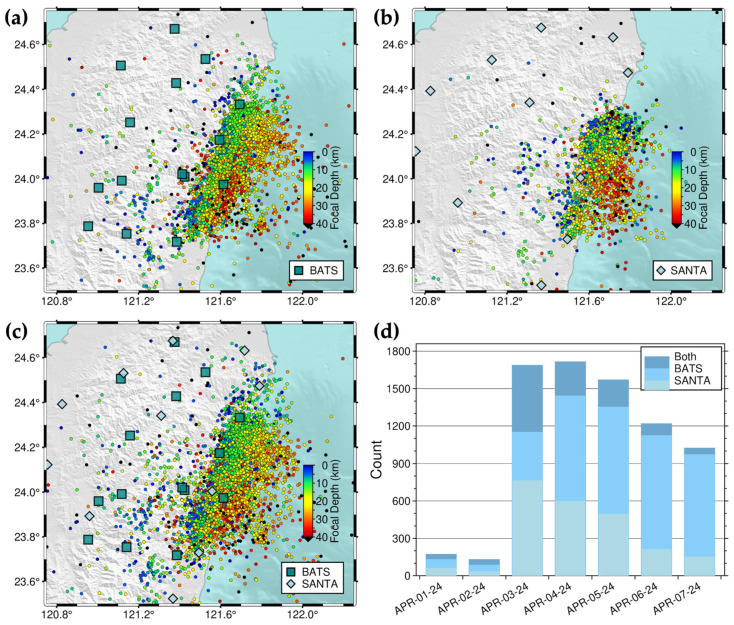
Station selection test of the 2024HL-RT-MEMS. One-week seismicity in the Hualien area with (**a**) BATS only, (**b**) SANTA only, and (**c**) both BATS and SANTA between 1 April 2024 and 7 April 2024. The blue-green squares and light-blue diamonds represent seismic stations of BATS and SANTA, respectively, and circles earthquakes color-coded by focal depths. (**d**) Daily earthquake numbers cataloged by the 2024HL-RT-MEMS of different station combinations. The date format is month-day-year, for example, APR-01-24 represents 1 April 2024.

We selected seismic stations within an 85 km radius of the mainshock epicenter, comprising 16 SANTA stations and 14 BATS stations. A comparison of one-week earthquake catalogs (1–7 April 2024) derived from continuous waveform data using different station configurations—14 BATS stations only, 16 SANTA stations only, and all 30 BATS and SANTA stations—revealed 6082, 2222, and 7275 seismic events, respectively (Figure 6). Given SANTA’s sparser station distribution compared to BATS in eastern Taiwan, the BATS-only configuration detected more seismic events than the SANTA-only configuration. The combined SANTA and BATS configuration yielded the highest number of detected seismic events.

#### 3.2.2. Earthquake Report of the 2024HL-RT-MEMS

**Figure 7:**
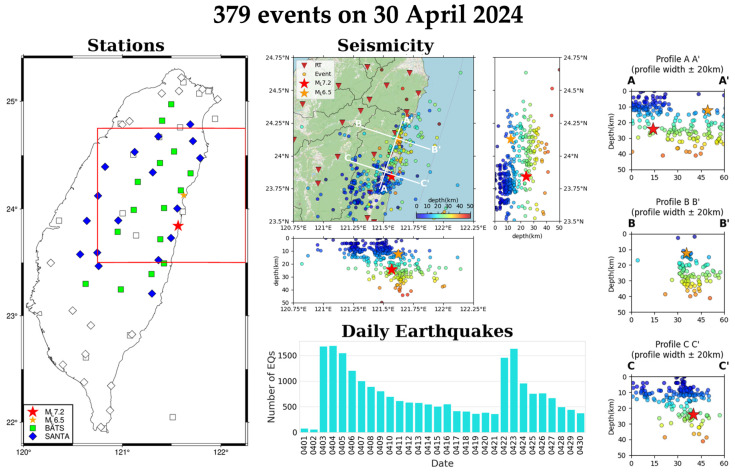
Daily earthquake report of 3 April 2024 *M*_L_ 7.2 Hualien earthquake sequence on 30 April 2024, including station map, map view, N-S and W-E cross-sections of daily seismicity, daily earthquake frequency for the preceding 30 days, and three more detailed cross-sections. In the Stations subplot, squares and diamonds represent seismic stations of BATS and SANTA, respectively, and the blue-green and light-blue coded ones are seismic stations used in the 2024HL-RT-MEMS. In the Seismicity subplot, the red inverted triangles represent seismic stations and circles earthquakes color-coded by focal depths. The date format in Daily Earthquakes subplot is month-day, for example, 0412 represents 12 April.

Daily earthquake reports for the 2024HL-RT-MEMS have been disseminated to researchers via email since 4 April 2024. Each report includes a station distribution map, a map view of daily seismicity with E-W and N-S profiles, and the daily number of earthquakes (Figure 7).

#### 3.2.3. Long-Term Seismicity of the 2024HL-RT-MEMS

**Figure 8:**
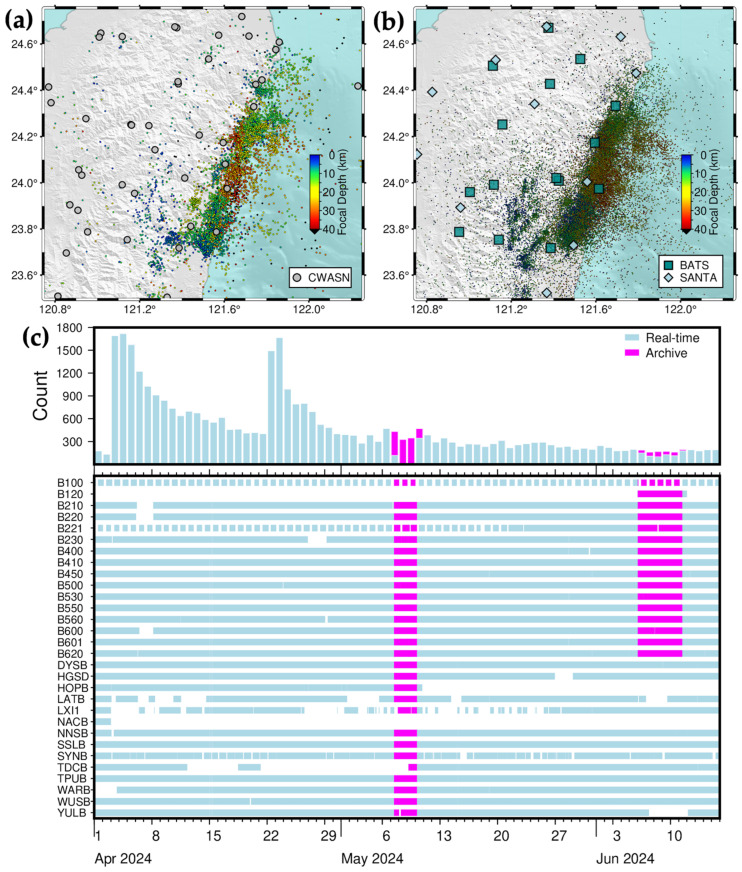
Long-term, two-and-half-month, seismicity in the Hualien area between 1 April 2024 and 15 June 2024. (**a**) 7499 earthquakes listed in the CWA catalog downloaded on 24 March 2025; (**b**) 33, 148 earthquakes listed in the 2024HL-RT-MEMS catalog issued on 16 June 2024. The gray circles, blue-green squares, and light-blue diamonds represent seismic stations of CWASN, BATS, and SANTA, respectively, and circles earthquakes color-coded by focal depths. (**c**) daily earthquake number (**upper panel**) and completeness of saved continuous waveform data (**lower panel**).

For the two-and-a-half-month period following the 2024 *M*_L_ 7.2 Hualien earthquake sequence, from 1 April 2024 to 15 June 2024, the CWA reported 7499 earthquakes (retrieved on 24 March 2025) (Figure 8a), while the 2024HL-RT-MEMS cataloged a total of 33,148 earthquakes (Figure 8b). The 2024HL-RT-MEMS experienced a shutdown period commencing on 7 May 2024 due to a compatibility issue with an older version of ObsPy [33,34], which caused data disconnections. Following an ObsPy update, data transmission was restored on 10 May 2024. During 6–10 June 2024, the transmission of continuous waveform data from SANTA to IES was interrupted due to an outage of the server at IES. These missing waveform data were subsequently retrieved from IES and NCREE’s data archive to ensure dataset continuity and earthquake catalog completeness (Figure 8c).

### 3.3. The Chia-Nan and the 2025 M_L_ 6.4 Dapu Earthquake Sequence (2025CN-RT-MEMS)

Following an *M*_L_ 5.2 earthquake in southwestern Taiwan on 29 December 2024 (19:51:36 UTC), we established an RT-MEMS, designated the 2025CN-RT-MEMS, to continuously monitor earthquake activity in the Chia-Nan (CN) area of southwestern Taiwan and issue daily earthquake reports via email. Three weeks later, this system recorded the unexpected *M*_L_ 6.4 Dapu earthquake sequence, which caused damage to over 2000 buildings and resulted in 50 injuries.

#### 3.3.1. Station Selection and Test of the 2025CN-RT-MEMS

**Figure 9:**
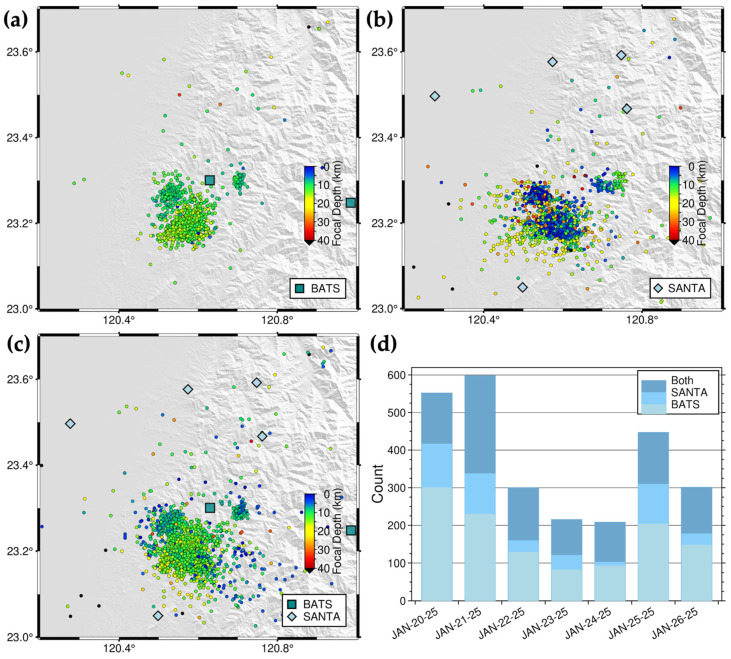
Station selection test of the 2025CN-RT-MEMS. One-week seismicity in the Chia-Nan area with (**a**) BATS only, (**b**) SANTA only, and (**c**) both BATS and SANTA between 20 January 2025 and 26 January 2025. The blue-green squares and light-blue diamonds represent seismic stations of BATS and SANTA, respectively, and circles earthquakes color-coded by focal depths. (**d**) Daily earthquake numbers listed by the 2025CN-RT-MEMS of different station combinations. The date format is month-day-year, for example, JAN-20-25 represents 20 January 2025.

Since April 2024, the Chia-Nan area has experienced 11 earthquakes with magnitudes greater than 5 (*M*5+). Consequently, the 2025CN-RT-MEMS was designed to monitor both the 2025 *M*_L_ 6.4 Dapu earthquake sequence and broader seismicity patterns in the Chia-Nan area. We selected seismic stations within an 85 km radius of the study area center, comprising ten SANTA stations and eight BATS stations. A comparison of one-week earthquake catalogs (20–26 January 2025) derived from continuous waveform data using different station configurations—eight BATS stations only, ten SANTA stations only, and all 18 BATS and SANTA stations—revealed 1099, 1573, and 2482 seismic events, respectively (Figure 9). Given BATS’s sparser station distribution compared to SANTA in western Taiwan, the SANTA-only configuration detected more seismic events than the BATS-only configuration. The combined SANTA and BATS configuration yielded the highest number of detected seismic events.

#### 3.3.2. Earthquake Report of the 2025CN-RT-MEMS

**Figure 10:**
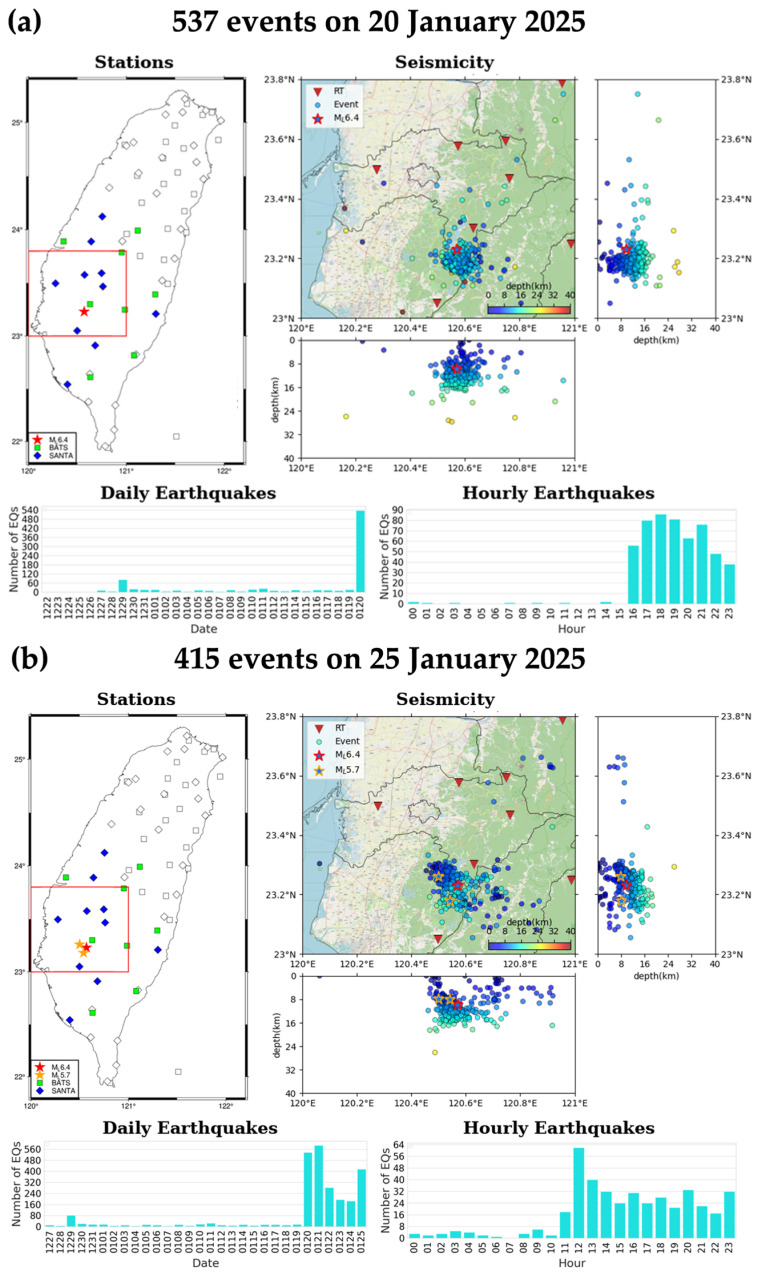
Daily earthquake reports of (**a**) *M*_L_ 6.4 Dapu mainshock on 20 January 2025 (16:17:26 UTC) and (**b**) two *M*_L_ 5.7 aftershocks on 25 January 2025 (11:49:17 UTC and 23:38:53 UTC), including station distribution, seismicity, and daily and hourly earthquake numbers. In the Stations subplots, squares and diamonds represent seismic stations of BATS and SANTA, respectively, and the blue-green and light-blue coded ones are seismic stations used in the 2025CN-RT-MEMS. In the Seismicity subplots, the red inverted triangles represent seismic stations and circles earthquakes color-coded by focal depths. The date format in the Daily Earthquakes subplots is month-day, for example, 0120 represents 20 January.

Daily earthquake reports for the 2025CN-RT-MEMS have been disseminated to researchers via email since 3 January 2025. Each report includes a station distribution map, a map view of daily seismicity with E-W and N-S profiles, and hourly and daily earthquake counts (Figure 10). Due to the lower number of aftershocks following the 2025 *M*_L_ 6.4 Dapu mainshock compared to the 2024 *M*_L_ 7.2 Hualien mainshock, we issued 6-h and daily earthquake reports to researchers via email.

#### 3.3.3. Long-Term Seismicity of the 2025CN-RT-MEMS

**Figure 11:**
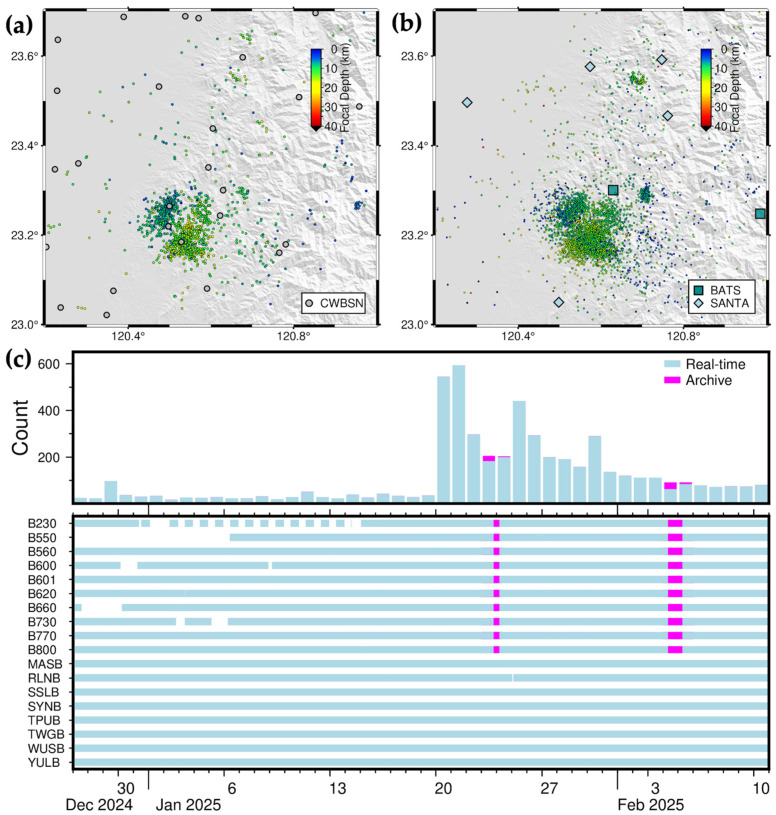
Long-term, two-and-half-month, seismicity in the Chia-Nan area between 17 December 2024 and 10 February 2025. (**a**) 1310 earthquakes listed in the CWA catalog downloaded on 24 March 2025; (**b**) 4294 earthquakes listed in the 2025CN-RT-MEMS catalog issued on 11 February 2025. The gray circles, blue-green squares, and light-blue diamonds represent seismic stations of CWASN, BATS, and SANTA, respectively, and circles earthquakes color-coded by focal depths. (**c**) daily earthquake number (**upper panel**) and completeness of saved continuous waveform data (**lower panel**).

During the 46-day period from 27 December 2024 to 10 February 2025, the 2025CN-RT-MEMS recorded 4294 earthquakes in the Chia-Nan area (Figure 11b), while the CWA reported 1310 earthquakes (retrieved on 24 March 2025) (Figure 11a). Continuous waveform data from SANTA was interrupted on two occasions during this period. Following communication with technical staff, it was determined that the server at IES receiving SANTA data had experienced an outage. After the necessary repairs were completed, data acquisition resumed, and archived data were obtained from IES and NCREE to ensure dataset continuity and earthquake catalog completeness (Figure 11c).

## 4. Discussion

We have established three stable real-time microearthquake monitoring systems in Taiwan through the technical integration of data transmission protocols, automated processing algorithms, and related earthquake parameter analyses. The month-long catalogs from the three RT-MEMS demonstrate similar seismic distributions and a greater number of earthquakes compared to the standard CWA catalog (Figure 5a,b, Figure 8a,b and Figure 11a,b). Furthermore, given the complexities associated with the routine maintenance of a nationwide seismic network, the combined networks of CSN, BATS, and SANTA, with station spacing on the order of tens of kilometers, represent a reliable and reasonable station configuration for the continuous observation of both regular background seismicity and aftershock sequences following moderate-to-large earthquakes.

In current research utilizing deep learning-based earthquake catalogs, direct application for interpreting seismogenic/seismotectonic structures is generally avoided (e.g., [55,56,57]). Among the most commonly employed deep learning models for phase picking, EQ-Transformer [40] and PhaseNet [41], PhaseNet has demonstrated superior predictive performance across tests using standard seismic datasets of varying scales from events at teleseismic and regional-to-local distances [58]. Furthermore, while PhaseNet potentially detects a lower number of earthquakes than EQ-Transformer, the earthquake catalog produced by PhaseNet may better delineate subsurface geological structures (e.g., [59]). Zhu et al. [60] applied their new phase association model, GaMMA, to extract an earthquake catalog of the 2019 Ridgecrest, California, earthquake sequence and compared four earthquake catalogs using the same waveform dataset but different algorithms and workflows. Zhu et al. [60] found that the four deep learning and template-matching catalogs—one using a deep learning phase picking model [61], one using both deep learning phase picking and association models [60], and two using a template-matching algorithm [20,62]—contain 1.5 to 2.9 times more earthquakes than the standard catalog, the Southern California Seismic Network (SCSN) catalog. However, the deep learning catalogs did not necessarily detect more seismic events than template-matching algorithms. These tests suggest that although a variety of deep learning models are available to significantly reduce the workload of data processing, optimal models and workflows should be adapted to specific tectonic settings and seismic behavior to best suit individual research topics [58].

In addition to testing different deep learning-based phase-picking and/or association models, researchers typically employ additional data processing procedures to refine earthquake events from deep learning earthquake catalogs. These procedures include absolute (e.g., NonLinLoc [63] and HypoSVI [64]) or relative (e.g., hypoDD [23] and GrowClust [65]) earthquake location methods, and even manual review. Consequently, there is a growing body of research dedicated to developing data processing workflows that incorporate deep learning models for earthquake catalog analysis (e.g., [11,27,66,67]). Sun et al. [27] used a two-month catalog of the 2022 *M*_L_ 6.9 Chihshang earthquake sequence extracted from the 2022CSN-RT-MEMS, developed in this study, and refined the catalog with manually checked phase arrivals, hypoDD relocation [23], local and moment magnitudes, and focal mechanism solutions for small earthquakes. This refined catalog contributes a high-resolution and precise seismicity record for seismotectonic interpretation, and further serves as a validation dataset for related research, such as surface rupture investigation [68], geodetic observation and source rupture modeling [69,70,71], and seismic energy scaling relationship studies [72]. Recently, Kuo-Chen et al. [73] extracted a two-week catalog of the 2025 *M*_L_ 6.4 Dapu earthquake sequence from the 2025CN-RT-MEMS, developed in this study, and applied NonLinLoc [63] with a local 3D seismic velocity model [74] to relocate the deep learning catalog. Kuo-Chen et al. [73] found that earthquake numbers were reduced by 40%, from 3893 to 2335, by hypoDD [23] without individual location errors but remained almost unchanged by NonLinLoc [63] with precise location errors, which can be used for subsequent data evaluation. We continue to test open-source deep learning models and programs of traditional algorithms to integrate them into our RT-MEMS for reliable source parameters and seismic characteristics, including event time, source location, earthquake magnitude, first-motion polarity, focal mechanism solution, 1D/3D absolute and relative relocation, stress change, and seismic velocity inversion.

Taiwan exhibits complex seismogenic environments due to its tectonic setting at an arc-continental convergent boundary. This results in a collision regime in southeastern Taiwan, monitored by the 2022CSN-RT-MEMS; a transition from collision to subduction in northeastern Taiwan, monitored by the 2024HL-RT-MEMS; and a fold-and-thrust belt in southwestern Taiwan, monitored by the 2025CN-RT-MEMS. However, this RT-MEMS has not been thoroughly tested in monitoring areas outside Taiwan, such as those with volcanic or induced seismicity, geothermal activity, and carbon capture and storage (CCS) sites. The phase-picking model, SeisBlue [39], implemented in this RT-MEMS, was trained with a regional seismic catalog in Taiwan. Therefore, to apply this RT-MEMS to detect seismic signals other than tectonic events, the phase picking model should be replaced or retrained with datasets containing the targeted signal.

## 5. Conclusions

We have established three RT-MEMS in Taiwan providing timely information on changes in seismic activity following major earthquakes, as well as long-term catalogs for researchers to conduct further studies on earthquake source parameters. These RT-MEMS encompass both background seismicity and aftershock sequences of moderate-to-large earthquakes, and integrate continuous data from stable, high-quality seismic networks, deep learning modules, and automated data processing workflows. Current RT-MEMS primarily focus on the identification and extraction of seismic events, providing high-resolution earthquake catalog compared to the standard CWA catalog, and we will continue to enhance this RT-MEMS to automatically extract a wider range of source parameters, such as earthquake magnitude, P-wave polarity, focal mechanism solutions, and improved 3D absolute and relative locations.

## Data Availability

The continuous seismic data can be applied from the Institute of Earth Sciences, Academia Sinica [28], and the National Center for Research on Earthquake Engineering [29].

## Abbreviations

The following abbreviations are used in this manuscript:

BATS	Broadband Array in Taiwan for Seismology
CSN	Chihshang Seismic Network
CWA	Central Weather Administration
CWASN	Central Weather Administration Seismic Network
IES	Institute of Earth Sciences, Academia Sinica
NCREE	National Center for Research on Earthquake Engineering
RT-MEMS	Real-Time MicroEarthquake Monitoring System
SANTA	Seismic Array of NCREE in Taiwan
